# The Impact of Educational Interventions on Nursing Students' Attitudes Towards End‐of‐Life Care: A Cross‐Sectional Study

**DOI:** 10.1002/nop2.70642

**Published:** 2026-06-28

**Authors:** Mayra Veronese, Cristiana Rago

**Affiliations:** ^1^ Laboratory of Studies and Evidence Based Nursing, Department of Cardiac, Thoracic, Vascular Sciences and Public Health University of Padua Padua Italy; ^2^ Department of Nursing and Midwifery Local Health Unit Tuscany Center Florence Italy

**Keywords:** end‐of‐life care, Frommelt Attitude Toward Care of the Dying Scale, nursing education, nursing students, palliative care

## Abstract

**Aim:**

To evaluate nursing students' attitudes towards end‐of‐life care and assess the impact of an elective educational course on shaping these attitudes.

**Design:**

A cross‐sectional observational study with a post‐test–only design was conducted at the University of Padua in northern Italy.

**Methods:**

The study involved 475 third‐year nursing students, with 228 participants (48%) completing an online survey. The validated Frommelt Attitude Toward Care of the Dying Scale, Form B, Italian Version (FATCOD Form B‐I) was used, and data were analysed through descriptive statistics, *t*‐tests and correlation analyses.

**Results:**

Participants demonstrated overall positive attitudes towards end‐of‐life care, with a mean FATCOD Form B‐I score of 118.65 (SD = 9.63). Female students scored significantly higher in communication (*p* < 0.001) and family care (*p* = 0.002) than males. However, no significant differences were found between students who completed the elective palliative care course and those who did not. Fear and discomfort with death negatively influenced relationship building with patients, while communication and active care were strongly correlated (*r* = 0.45, *p* < 0.001).

**Conclusion:**

Although nursing students exhibited generally positive attitudes, the elective course alone was insufficient to significantly enhance preparedness for end‐of‐life care. A comprehensive, integrated palliative care education programme is necessary to address emotional barriers and improve relational competencies, ensuring students are equipped for compassionate and competent end‐of‐life care.

**Implications for the Profession:**

Although nursing students exhibited generally positive attitudes towards end‐of‐life care, theoretical instruction alone proved insufficient to significantly enhance their preparedness for palliative care practice. These findings have direct implications for nursing education and clinical care quality: integrating comprehensive, experiential palliative care education throughout nursing curricula, rather than relying on optional standalone courses, may better equip future nurses to deliver compassionate and competent end‐of‐life care.

**Impact:**

- Despite international recommendations, many nursing students feel unprepared for end‐of‐life care. This study examined whether an elective palliative care course produces meaningful attitudinal change among final‐year nursing students.
- Students showed generally positive attitudes, with the highest scores in Family care and Communication. No significant difference emerged between students who attended the elective course and those who did not.
- The findings impact nursing educators and curriculum designers, ultimately benefiting future nurses and the terminally ill patients and families they will care for.

**Reporting Method:**

This study was reported in accordance with the Strengthening the Reporting of Observational Studies in Epidemiology (STROBE) guidelines for observational studies.

**Patient or Public Contribution:**

No patient or public contribution.

## 1 Introduction

In recent years, the significance of palliative care within healthcare systems has grown substantially. Factors such as the COVID‐19 pandemic, an ageing population and an increasing prevalence of chronic and severe illnesses have intensified the demand for palliative care services. Addressing these challenges necessitates integrating general palliative care competencies into nursing education from the undergraduate level onward (Parekh de Campos et al. 2022). Providing palliative care, particularly to terminally ill patients, can impose considerable psychological strain on nurses. They may experience feelings of helplessness, nervousness, guilt, regret, sadness, anxiety, frustration, anger and even repulsion (Lo Iacono et al. 2024; Kostka et al. 2021; Nabirye et al. 2025). This stress is compounded by the notion that caring for dying individuals is not an innate human inclination. For nursing students, these challenges often manifest as heightened emotional distress, including fear of their reactions to death, loss of control and as perceived inability to offer adequate and compassionate support to patients and their families. Such emotions can lead to negative attitudes towards end‐of‐life care (Lo Iacono et al. 2024).

The World Health Organization (WHO) underscores the need for foundational palliative care training for all healthcare professionals (World Health Organization 2020). Despite recommendations from international bodies, such as the European Association for palliative care, and educational efforts aimed at improving nursing students' competencies (Hökkä et al. 2020; Pereira et al. 2021), many still feel unprepared to meet the complex needs of terminally ill patients (Heath et al. 2022; Serafin et al. 2025). This sense of inadequacy is not only linked to the emotional challenges inherent in end‐of‐life care but also to the inconsistent and often fragmented integration of palliative care training within curricula. Such disparities exist both across and within countries (Yoong et al. 2023).

The attitude of nursing students towards death and palliative care is a critical area of inquiry, particularly as these future healthcare professionals will play a pivotal role in managing end‐of‐life care. Nursing students' attitudes are shaped by various factors, including educational experiences, personal encounters with death and the quality of training received during their studies. Nursing students often experience significant anxiety regarding death, which can adversely affect their attitudes towards palliative care (Xu and Yu 2024). Educational interventions have been shown to positively influence nursing students' attitudes towards death and palliative and end‐of‐life care. These findings emphasize the importance of integrating specific educational content into nursing curricula to foster more positive and supportive attitudes among students (Mastroianni et al. 2015; Edo‐Gual et al. 2018). The End‐of‐Life Nursing Education Consortium (ELNEC) curriculum has been particularly effective in enhancing nursing students' knowledge and attitudes towards end‐of‐life care, demonstrating that structured educational programmes can significantly improve students' preparedness for palliative care (Ferrell et al. 2018; Kannappan 2025).

In Italy, the enactment of Law 38/2010 represented a significant milestone in formally recognizing and implementing palliative care and pain management as fundamental elements of the national healthcare system. This legislation underscores the right of every individual to receive adequate pain management and palliative care, reflecting a broader societal commitment to improving end‐of‐life care. The law also emphasizes the importance of creating and maintaining care networks that can provide continuous and coordinated care, tailored to the specific needs of patients and their families.

For instance, Xu and Yu (2024) found a negative correlation between death anxiety and attitudes towards palliative care among undergraduate nursing students, suggesting that heightened fear of death may hinder their willingness to engage in palliative and end‐of‐life care (Xu and Yu 2024). This aligns with the findings of Kim and Hwang (2014), who noted that nurses' attitudes towards death significantly influence their preparedness to practice palliative care, indicating that a positive orientation towards death is essential for effective palliative care delivery (Kim and Hwang 2014). Several studies have shown that nursing students who participate in courses focused on death and dying exhibit more favourable attitudes towards these topics. For example, Özveren et al. (2022) highlighted that students who were exposed to storytelling techniques showed improved perceptions of death and more positive attitudes (Özveren et al. 2022). Similarly, Zhou et al. (2021) found that prior exposure to death education correlated with more positive attitudes towards palliative care among nursing students (Zhou et al. 2021). These findings underscore the necessity of incorporating death education into nursing programmes to better prepare students for the realities of palliative care. In summary, the relationship between nursing students' attitudes towards death and their engagement with palliative care is complex and influenced by educational experiences. The integration of targeted courses on palliative and end‐of‐life care within nursing curricula may not only alleviate death anxiety but also foster a more compassionate and competent approach to palliative care among future nurses (Durojaiye et al. 2023). Accordingly, this study aims to investigate nursing students' attitudes towards providing care to dying patients and to assess the impact of an elective educational course within bachelor's degree programmes on shaping these attitudes by the end of their studies.

## 2 Materials and Methods

### 2.1 Study Design

A cross‐sectional observational study with a post‐test–only design was conducted using a self‐administered, validated online scale. This study was reported according to the ‘STrenthening the Reporting of Observational Studies in Epidemiology’ (STROBE) guidelines (von Elm et al. 2008).

### 2.2 Participants and Procedures

This study involved third‐year students enrolled in a bachelor's degree programme in nursing at the University of Padua in northern Italy (*N* = 475).

During the 3‐year programme, students acquire skills to provide nursing care in hospital, residential, home and outpatient settings. Additionally, there are optional activities that students can choose to participate in to enhance their knowledge in specific areas as well as preclinical labs that address distinct topics. Among the topics offered by the university is ‘End‐of‐life care: quality of life and family support’, which also includes the following content: the process of terminal illness, the treatment of typical symptoms in the terminal phase, the relationship with the person and the family during the terminal stage of life, the management of anticipatory grief and bereavement, and the ethical aspects of managing a person in the terminal phase. The palliative care course consisted of 30 h of theoretical instruction, delivered in‐person through six lectures of 5 h each during the third year of the programme, with practical experience acquired during students' clinical placements in both hospital and community‐based settings. Not all students chose to attend the same elective palliative care course; specifically, not all of them participated in the elective course on ‘End‐of‐life care: quality of life and family support’.

### 2.3 Instruments

The survey, administered only after students completed the elective palliative care course, included two sections: (a) a demographic section and (b) the Frommelt Attitude Toward Care of the Dying Scale Form B, Italian Version (FATCOD Form B‐I).

- The demographic section collected data on participants' gender, age and whether they had attended the elective course ‘End‐of‐life care: quality of life and family support’.
- Attitudes towards the care of dying patients were assessed using the FATCOD Form B‐I. The Italian validated version was used, as it has been previously tested in nursing students and shown to be reliable and appropriate for the Italian cultural context (Mastroianni et al. 2015). The instrument consists of 30 items rated on a 5‐point Likert scale, ranging from 1 (strong disagreement) to 5 (strong agreement) with total scores ranging from 30 to 150. Higher scores indicate more positive attitudes towards the care of dying patients. The scale includes both positively and negatively worded items intended to capture multiple dimensions of attitudes towards end‐of‐life care. Negatively worded items were reverse‐scored prior to analysis, in accordance with the scoring procedures described in the Italian validation study (Mastroianni et al. 2015), so that higher scores consistently reflect more positive attitudes. Unlike the original FATCOD Form B, which is treated as a unidimensional scale, the FATCOD Form B‐I identifies six dimensions through factorial analysis: ‘Fear/Malaise’, ‘Communication’, ‘Relationship’, ‘Care of the Family’, ‘Family as Caring’ and ‘Active Care’. These reflect specific areas of student attitudes relevant to end‐of‐life care education (Mastroianni et al. 2015). The FATCOD Form B‐I has shown good psychometric properties in Italian nursing students, including strong internal consistency (Cronbach's alpha = 0.81), test–retest reliability and construct validity. It is widely used in Italian research to assess educational impact on end‐of‐life care attitudes (Mastroianni et al. 2015). It should be noted that multiple versions and cultural adaptations of the FATCOD instrument exist, which may differ in item wording, classification and factor structure. To ensure methodological consistency, this study adhered to the scoring procedures defined in the Italian validation (Mastroianni et al. 2015).

### 2.4 Data Collection

The survey was uploaded and distributed via Microsoft Form between 15 February and 15 April 2022. Eligible participants were identified as third‐year nursing students enrolled in the bachelor's degree programme at the participating university.

The institutional email addresses of eligible students were provided by the degree programme coordinator, following formal authorization from the university's academic committee. This ensured that the contact procedure complied with institutional policies and ethical standards.

The study objectives were communicated to participants through an email from the researchers, who also offered additional information upon request. Participants completed the self‐administered survey voluntarily after providing informed consent. Four reminder emails were sent to participants who had not yet completed the survey.

### 2.5 Data Analysis

Descriptive statistics were used including calculation of central tendency (mean [*M*] and median), variability (standard deviation [SD]) and frequencies and percentages for categorical data.

Pearson's correlation coefficients (*r*) were calculated to explore relationships among the FATCOD Form B‐I dimensions, with significance determined by *p*‐values. The strength of the correlation was interpreted as follows: weak (0.10 ≤ ∣*r*∣ < 0.30), moderate (0.30 ≤ ∣*r*∣ < 0.50) and strong (∣*r*∣ ≥ 0.50). The direction (positive or negative) indicated whether the variables increased together or inversely, respectively. Statistical significance was determined using *p*‐values (*p* < 0.05 as the threshold) (de Winter et al. 2016).

Independent samples *t*‐tests were conducted to evaluate differences in mean FATCOD Form B‐I scores and subdimensions based on demographic variables (e.g., gender, participation in elective courses). A two‐sided *p*‐value of < 0.05 was considered statistically significant in the analysis. All analyses were performed using SPSS software, v27 (IBM Corp., Armonk, NY).

### 2.6 Ethical Statement

This study adhered to the ethical standards set forth in the Declaration of Helsinki. Compliance with data protection regulations was ensured under Regulation 2016/679 of the European Parliament (GDPR) and other applicable laws on personal data protection. The research received approval from the Academic Committee on 20 January 2022, following a thorough assessment of all ethical considerations. Given the non‐interventional nature of the study and in line with Italian regulations, no external Ethics Committee approval was required. Before participating, all individuals received comprehensive information about the study's aims, procedures and data management policies, and then gave their written informed consent. To safeguard participants' anonymity, all questionnaires were anonymous, ensuring non‐identifiability. Data collection, storage and analysis were conducted in compliance with strict confidentiality standards, with access restricted exclusively to the research team.

To minimize potential power relations between researchers and participants, the survey was distributed by an external researcher who had no teaching role or academic authority over the students. This ensured that students' decision to participate was free from any perceived pressure or influence. Furthermore, no personal data were collected, and participation or non‐participation had no consequences for students' academic standing.

## 3 Results

### 3.1 Characteristics of the Sample

A total of 228 students (48% of the population) responded to the survey, with the majority being female (*N* = 189; 82.9%) and an average age of 23.3 years, with the range 20–43 years. Of the participants, 71% (*N* = 162) had completed an elective course titled ‘End‐of‐life care: quality of life and family support’, highlighting their exposure to structured palliative care education. Participants' attitudes towards care for the dying were assessed post‐test using the FATCOD Form B‐I. The total mean score on the FATCOD Form B‐I scale was 118.65 (SD = 9.63), indicating generally positive attitudes towards end‐of‐life care (Table 1).

**TABLE 1:** Characteristics of the participant (*N* = 228).

Characteristics	
Gender, *n* (%)	
Female	189 (82.9)
Male	39 (17.1)
Age	
Mean (SD)	23.3 (3.9)
Median (min–max)	22 (20–43)
Participation an elective course^a^, *n* (%)	
Yes	162 (71)
No	66 (29)
Total value FATCOD Form B‐I	
Mean (SD)	118.65 (9.63)
Median (min–max)	118 (97–144)

^a^ Elective course ‘End‐of‐life care: quality of life and family support’.

### 3.2 Frommelt Attitudes Toward Care of the Dying Scale, Form B, Italian Version

Table 2 presents the median values of individual items. Item‐specific analysis revealed strong agreement with statements such as ‘Giving care to the dying person is a worthwhile experience’ (*M* = 4.55, SD = 0.61) and ‘Families need emotional support to accept the behaviour changes of the dying person’ (*M* = 4.57, SD = 0.55). Conversely, there was notable disagreement with items like ‘The dying person should not be allowed to make decisions about his or her physical care’ (*M* = 1.64, SD = 0.80) and ‘Educating families about death and dying is not a non‐family caregiver's responsibility’ (*M* = 1.65, SD = 0.84).

**TABLE 2:** The mean value and standard deviation of all items of the Frommelt Attitudes Toward Care of the Dying Scale (FATCOD) Form B‐I.

Questions	Mean^a^ (SD)
1. Giving care to the dying person is a worthwhile experience	4.55 (0.61)
2. Death is not the worst thing that can happen to a person	3.61 (1.05)
3. I would be uncomfortable talking about impending death with the dying person	3.04 (0.96)
4. Caring for the patient's family should continue throughout the period of grief and bereavement	4.36 (0.8)
5. I would not want to care for a dying person	1.88 (0.89)
6. The non‐family caregivers should not be the one to talk about death with the dying person	1.99 (0.86)
7. The length of time required to give care to a dying person would frustrate me	2.09 (1)
8. I would be upset when the dying person I was caring for gave up hope of getting better	3.01 (0.97)
9. It is difficult to form a close relationship with the dying person	2.26 (0.96)
10. There are times when death is welcomed by the dying person	4.03 (0.62)
11. When a patient asks, ‘Am I dying?’ I think it is best to change the subject to something cheerful	1.98 (0.89)
12. The family should be involved in the physical care (feeding, personal hygiene) of the dying person	4.39 (0.64)
13. I would hope the person I'm caring for dies when I am not present	2.47 (0.94)
14. I am afraid to become friends with a dying person	2.55 (1.15)
15. I would feel like running away when the person actually died	2.08 (0.94)
16. Families need emotional support to accept the behavior changes of the dying person	4.57 (0.55)
17. As a patient nears death, the non‐family caregiver should withdraw from his or her involvement with the patient	2.48 (1.05)
18. Families should be concerned about helping their dying member make the best of his or her remaining life	4.55 (0.64)
19. The dying person should not be allowed to make decisions about his or her physical care	1.64 (0.8)
20. Families should maintain as normal an environment as possible for their dying member	4.32 (0.64)
21. It is beneficial for the dying person to verbalize his or her feelings	4.25 (0.71)
22. Care should extend to the family of the dying person	4.49 (0.6)
23. Caregivers should permit dying persons to have flexible visiting schedules	4.37 (0.65)
24. The dying person and his or her family should be the in‐charge decision makers	3.96 (0.75)
25. Addiction to pain relieving medication should not be a concern when dealing with a dying person	3.49 (1.13)
26. I would be uncomfortable if I entered the room of a terminally ill person and found him/her crying	2.68 (0.99)
27. Dying persons should be given honest answers about their condition	4.21 (0.74)
28. Educating families about death and dying is not a non‐family caregiver's responsibility	1.65 (0.84)
29. Family members who stay close to a dying person often interfere with the professional's job with the patient	2.88 (0.96)
30. It is possible for non‐family caregivers to help patients prepare for death	4.23 (0.79)

Abbreviation: SD, standard deviation.

^a^ On a Likert scale ranging from 1 (‘strongly disagree’) to 5 (‘strongly agree’).

As shown in Table 3, the highest average values were observed in the dimensions of ‘The Care of the Family’ (*M* = 4.47; SD = 0.51) and ‘Family as caring’ (*M* = 4.42; SD = 0.49). Following by dimensions of ‘Communication’ (*M* = 4.07; SD = 0.48) and ‘Active care’ (*M* = 4.04; SD = 0.48). Correlation analysis revealed key relationships among dimensions of the FATCOD Form B‐I scale. Stronger attitudes towards ‘Active care’ were positively associated with better ‘Communication’ (*r* = 0.45, *p* < 0.001) and ‘The Care of the Family’ (*r* = 0.32, *p* < 0.001). A significant relationship was also observed between ‘Fear/Malaise’ and ‘Relationship‐building’ (*r* = 0.29, *p* < 0.001), suggesting that discomfort or anxiety around end‐of‐life care may hinder forming meaningful connections with patients.

**TABLE 3:** Pearson correlation results between dimensions of the Frommel Attitudes Toward Care of the Dying Scale (FATCOD) Form B‐I.

Dimensions	Mean^a^ (SD)	Fear/Malaise		The Care of the Family		Communication		Family as caring		Relationship		Active care	
		Pearson *r*	*p*	Pearson *r*	*p*	Pearson *r*	*p*	Pearson *r*	*p*	Pearson *r*	*p*	Pearson *r*	*p*
Fear/Malaise	3.64 (0.55)	1	—										
The Care of the Family	4.47 (0.51)	0.39	0.708	1	—								
Communication	4.07 (0.48)	0.25	< 0.001	0.34	< 0.001	1	—						
Family as caring	4.42 (0.49)	0.12	0.070	0.34	< 0.001	0.29	< 0.001	1	—				
Relationship	3.73 (0.46)	0.29	< 0.001	0.31	< 0.001	0.38	< 0.001	0.27	< 0.001	1	—		
Active care	4.04 (0.48)	0.11	0.109	0.32	< 0.001	0.45	< 0.001	0.22	< 0.001	0.22	< 0.001	1	—

Abbreviation: SD, standard deviation.

^a^ A mean score was computed by summing the scores of the items within each category and dividing it by the total number of factors within that category. Fear/Malaise: Questions 1, 3, 5, 7, 8, 13, 14, 15, 26; The Care of the Family: Questions 4,16, 22; Communication: Questions 2, 6, 11, 27, 28, 30; Family as caring: Questions 12,18, 20; Relationship: Questions 9, 10, 17, 21, 29; Active Care: Questions 19, 23, 24, 2.

Gender differences emerged as significant in several dimensions (Table 4). Female participants demonstrated higher overall FATCOD Form B‐I scores (*M* = 119.44, SD = 9.65) compared to males (*M* = 114.82, SD = 8.65), and this difference was statistically significant (*p* = 0.006). Females reported more positive attitudes towards the dimensions: ‘The Care of the Family’ (female: *M* = 4.52 vs. male: *M* = 4.24; *p* = 0.002) and ‘Communication’ compared to males (female: *M* = 4.12; male: *M* = 3.81; *p* < 0.001).

**TABLE 4:** Differences in Frommelt Attitudes Toward Care of the Dying Scale (FATCOD) Form B‐I and between variables.

Variable	Total FATCOD‐B‐I		Fear/Malaise		The Care of the Family		Communication		Family as caring		Relationship		Active care	
	Mean (SD)	*p*	Mean (SD)	*p*	Mean (SD)	*p*	Mean (SD)	*p*	Mean (SD)	*p*	Mean (SD)	*p*	Mean (SD)	*p*
Gender														
Female	119.44 (9.65)	0.006	3.65 (0.55)	0.511	4.52 (0.48)	0.002	4.12 (0.45)	< 0.001	4.43 (0.48)	0.288	3.75 (0.47)	0.174	4.07 (0.48)	0.117
Male	114.82 (8.65)		3.58 (0.55)		4.24 (0.58)		3.81 (0.51)		4.34 (0.54)		3.64 (0.41)		3.93 (0.46)	
Elective course														
Yes	118.49 (9.46)	0.696	3.64 (0.56)	0.916	4.48 (0.50)	0.641	4.04 (0.49)	0.246	4.44 (0.45)	0.333	3.73 (0.43)	0.786	4.02 (0.48)	0.190
No	119.04 (10.09)		3.63 (0.54)		4.44 (0.54)		4.13 (0.45)		4.37 (0.58)		3.74 (0.52)		4.11 (0.46)	

## 4 Discussion

The present study examined nursing students' attitudes towards end‐of‐life care and assessed the impact of a dedicated elective course within bachelor's degree programmes on shaping these attitudes. This study provides important insights into nursing students' attitudes towards end‐of‐life care, measured using the FATCOD Form B‐I scale. Overall, participants demonstrated generally positive attitudes, reflecting a good level of preparedness for end‐of‐life care. The average score reflects a predisposition and affinity for providing care to terminally ill patients among third‐year nursing students.

The highest mean scores were observed in dimensions related to family involvement, specifically ‘Care of the Family’ and ‘Family as Caring’ as well as in ‘Communication’. As highlighted by Saarinen et al. (2023), family participation in hospital‐based palliative care is crucial and should be facilitated by offering opportunities for involvement in patient care and accommodating their presence in the hospital setting. This approach requires developing specific competencies not only for patient care but also for supporting families in collaboration with the healthcare team. According to Mastroianni et al. (2015), family involvement in palliative care should be addressed from two perspectives: providing support to the family, particularly during the grieving process, and involving relatives and significant others in the care of terminally ill patients to address their physical, clinical, spiritual, social and psychological needs. Furthermore, communication emerged as a significant dimension in the attitudes of nursing students. Effective communication is a cornerstone of palliative care, enabling nurses to build trust, convey empathy and address the needs of both patients and their families. Hökkä et al. (2024) outlined 10 essential competencies for evidence‐based palliative care, forming a comprehensive framework for high‐quality, patient‐centred practice. These competencies include: Knowledge of the fundamental characteristics of palliative care; Decision‐making skills and facilitation of the palliative care process; Expertise in symptom management; The ability to provide holistic patient‐centred support; Active communication focused on the patient and their family; Empathy in palliative care contexts; Spiritual sensitivity and competencies; Understanding of ethical and legal issues; Teamwork skills in an interdisciplinary setting; and Self‐awareness and reflective practice. These competencies provide a critical framework to ensure high‐quality, patient‐centred care in palliative settings. As Jeffers (2018) points out, nursing students are expected to achieve proficiency in caring for dying patients as a fundamental part of their training.

Although several previous studies (Abu‐El‐Noor and Abu‐El‐Noor 2016; Dobrowolska et al. 2019; Ferri et al. 2021; Krezel et al. 2025) did not identify significant gender differences in attitudes towards end‐of‐life care, the present findings showed that female students reported significantly higher overall FATCOD Form B‐I scores compared to male students (*p* = 0.006). These results are consistent with a larger body of evidence, including a meta‐analysis of 26 cross‐sectional studies with 9749 nursing students from 13 countries, which reported lower mean scores among male students in both knowledge and attitudes regarding end‐of‐life care (Wang et al. 2022). This suggests that, when evidence is considered across a broader range of studies, gender differences in attitudes towards end‐of‐life care may indeed be present. Analysis of specific dimensions further showed that female participants reported more positive attitudes in areas such as ‘Care of the Family’ and ‘Communication’, supporting the overall findings. These findings align with prior research suggesting that female nursing students may exhibit higher levels of empathy and more favourable attitudes in caregiving contexts, likely influenced by gendered socialization patterns (Atta et al. 2024; Gohri et al. 2025).

Low scores were observed in ‘Fear/Malaise’ and ‘Relationship’ dimensions. These findings suggest that students may feel less confident in managing the anxiety or emotional discomfort associated with caring for terminally ill patients, as well as in building meaningful relationships with patients and their families. The ‘Fear/Malaise’ dimension may reflect some difficulty in confronting the topic of death and the emotions connected to it, an area that could be further explored in educational contexts (Zhou et al. 2021). The low score in the Relationship dimension may indicate a need to develop stronger relational skills, which are crucial for providing empathetic and professional support to terminally ill patients (Suikkala et al. 2021).

Interestingly, no significant differences in overall FATCOD Form B‐I scores were observed between students who participated in the elective palliative care course and those who did not. These findings suggest that theoretical instruction alone, while valuable, may not be sufficient to produce measurable changes in attitudes towards end‐of‐life care. A more systematic integration of palliative care education throughout the nursing curriculum may be necessary to effectively influence students' perceptions and competencies. Notably, a study by Frommelt (2003) involving 115 American undergraduate nursing students reported a significant improvement in attitudes following participation in an educational programme centred on the care of terminally ill patients and their families. Similarly, Mastroianni et al. (2021), in a multicentre cross‐sectional study conducted in Italy, found that higher FATCOD Form B‐I scores were significantly associated with training in palliative care and prior experience with terminally ill patients. Furthermore, as highlighted in a systematic review by Jeong et al. (2020), the FATCOD Form B scale is frequently used to assess nurses' attitudes towards caring for terminally ill patients. Higher scores were observed among participants with advanced education, extensive experience or specific training in palliative care.

As described above, the palliative care course consisted of 30 h of theoretical instruction, with practical experience acquired during the students' clinical placements. However, it is important to note that this investigation was conducted among third‐year nursing students during the COVID‐19 pandemic, a period that likely influenced the results by altering the educational environment and limiting students' exposure to traditional clinical experiences.

These findings highlight the importance of complementing theoretical content with more engaging and experiential learning strategies. Several educational approaches could be considered to enhance the impact of palliative care courses, such as group reflection, simulation exercises, role‐playing, case‐based learning and interdisciplinary discussions. These experiential learning activities would ensure a more systematic and equitable exposure to end‐of‐life care scenarios, rather than relying solely on the variability of clinical placements, where encounters with dying patients cannot be guaranteed. Such an integrated approach, combining theoretical knowledge with experiential and reflective learning, would more effectively address the emotional barriers identified in this study, particularly in the dimensions of ‘Fear/Malaise’ and ‘Relationship’, and better equip nursing students for compassionate and competent end‐of‐life care. Integrating educational strategies that address the management of death‐related emotions and strengthening relational competencies is fundamental for delivering high‐quality palliative care (Agustina et al. 2025; Rosa et al. 2021; Kezar et al. 2025).

### 4.1 Strengths and Limitations

This study offers several strengths, the use of a validated tool for measuring attitudes (FATCOD Form B‐I), and the inclusion of final‐year nursing students, providing a relevant perspective close to the point of professional entry. These elements contribute to the depth and relevance of the findings.

However, some limitations should be acknowledged. The use of convenience sampling, although reflective of the target population, limits the generalizability of the results beyond the included settings. Moreover, the high non‐response rate may introduce a self‐selection bias, as those more interested or confident in end‐of‐life care may have been more likely to participate, thus potentially skewing the findings. This limits the external validity of the results and should be considered when interpreting the data.

Additionally, reliance on self‐reported measures carries the risk of social desirability bias, where students may overestimate their competencies or report more favourable attitudes than they hold. While the FATCOD Form B‐I scale has been widely used, it has also been critiqued for not capturing behavioural aspects of care or situational complexity (Edo‐Gual et al. 2018; Berndtsson et al. 2019). Its psychometric properties may vary across contexts and languages, and certain items may reflect outdated or culturally specific views.

A further limitation relates to the FATCOD instrument itself. Multiple versions and cultural adaptations of the scale exist, which differ in item wording, classification and underlying factor structure. While the original FATCOD Form B was conceptualized as a unidimensional scale, the Italian version (FATCOD Form B‐I) identifies multiple dimensions, including Fear/Malaise, Communication, Relationship, Care of the Family, Family as Caring and Active Care. This multidimensional structure limits direct comparability with studies using the original version or other adaptations. In addition, discrepancies in the classification and wording of certain items in the Italian version compared with the original FATCOD‐B may represent a potential source of measurement bias. Although the present study adhered to the scoring procedures defined in the Italian validation (Mastroianni et al. 2015), these differences should be considered when interpreting the findings and comparing results across studies.

The cross‐sectional design prevents tracking changes in attitudes over time, and data collection during the COVID‐19 pandemic—when clinical experiences were limited or modified—may have further impacted students' exposure to palliative care situations. Additionally, the post‐test–only design limits the ability to assess changes in attitudes over time and does not account for students' baseline attitudes prior to the educational experience.

Finally, the study focused on a single region and educational institution, limiting the cultural and contextual diversity of the sample. Future studies should include longitudinal designs, multiple institutions and objective assessments of palliative care competencies. Exploring factors such as personal spirituality or previous exposure to dying patients could also enhance understanding of students' attitudes.

## 5 Conclusion

This study provides valuable insights into the attitudes of nursing students towards palliative care and highlights areas for improvement in nursing education. The findings indicate generally positive attitudes among third‐year nursing students, particularly in the dimensions of ‘Care of the Family’, ‘Family as Caring’ and ‘Communication’. Significant emotional and educational barriers persist, particularly regarding fear and discomfort associated with death. Addressing these challenges requires a multifaceted approach, including integrating comprehensive palliative care content into nursing curricula and providing targeted interventions to build emotional resilience.

These findings suggest that nursing educators should move beyond standalone elective courses, embedding palliative care education throughout the nursing curriculum and incorporating experiential learning strategies, such as simulation, role‐playing and reflective practice, to more effectively shape students' attitudes towards end‐of‐life care.

Addressing the emotional barriers identified in this study, particularly in the dimensions of ‘Fear/Malaise’ and ‘Relationship’, requires targeted interventions aimed at building emotional resilience and strengthening relational competencies. Notably, gender differences were observed in specific dimensions, with female students demonstrating more positive attitudes in ‘Care of the Family’ and ‘Communication’. These findings align with prior research and emphasize the importance of tailoring educational strategies to address individual and group differences among students. By equipping nursing students with the skills and attitudes necessary for palliative care, educational institutions can better prepare future nurses to meet the growing demand for compassionate and competent end‐of‐life care.

## Funding

The authors have nothing to report.

## Ethics Statement

This study was approved by the Academic Committee of University of Padua on 20 January 2022. The study adhered to the ethical standards set forth in the Declaration of Helsinki and complied with Regulation 2016/679 of the European Parliament (GDPR).

## Consent

All participants provided written informed consent prior to participation. Participants were informed that their data would be anonymized and used solely for research purposes. Participation was entirely voluntary and had no consequences for students' academic standing.

## Conflicts of Interest

The authors declare no conflicts of interest.

## Data Availability

The data that support the findings of this study are available from the corresponding author upon reasonable request.
